# Gut microbiota dysbiosis in hyperuricaemia promotes renal injury through the activation of NLRP3 inflammasome

**DOI:** 10.1186/s40168-024-01826-9

**Published:** 2024-06-21

**Authors:** Xinghong Zhou, Shuai Ji, Liqian Chen, Xiaoyu Liu, Yijian Deng, Yanting You, Ming Wang, Qiuxing He, Baizhao Peng, Ying Yang, Xiaohu Chen, Hiu Yee Kwan, Lin Zhou, Jieyu Chen, Xiaoshan Zhao

**Affiliations:** 1 Zhujiang Hospital of Southern Medical University, Guangzhou, 510280 China; 2 School of Traditional Chinese Medicine, Southern Medical University, Guangzhou, 510515 China; 3 Dongguan Hospital of Guangzhou University of Chinese Medicine, Dongguan, 523000 China; 4 School of Chinese Medicine, Hong Kong Baptist University, Hong Kong, China; 5 Nanfang Hospital of Southern Medical University, Guangzhou, 510515 China

**Keywords:** Hyperuricaemia, Gut-kidney axis, Renal injury, Microbiota, Gut-derived uremic toxins, NLRP3 inflammasome

## Abstract

**Background:**

The prevalence of hyperuricaemia (HUA), a metabolic disorder characterized by elevated levels of uric acid, is on the rise and is frequently associated with renal injury. Gut microbiota and gut-derived uremic toxins are critical mediators in the gut-kidney axis that can cause damage to kidney function. Gut dysbiosis has been implicated in various kidney diseases. However, the role and underlying mechanism of the gut microbiota in HUA-induced renal injury remain unknown.

**Results:**

A HUA rat model was first established by knocking out the uricase (UOX). HUA rats exhibited apparent renal dysfunction, renal tubular injury, fibrosis, NLRP3 inflammasome activation, and impaired intestinal barrier functions. Analysis of 16S rRNA sequencing and functional prediction data revealed an abnormal gut microbiota profile and activation of pathways associated with uremic toxin production. A metabolomic analysis showed evident accumulation of gut-derived uremic toxins in the kidneys of HUA rats. Furthermore, faecal microbiota transplantation (FMT) was performed to confirm the effects of HUA-induced gut dysbiosis on renal injury. Mice recolonized with HUA microbiota exhibited severe renal injury and impaired intestinal barrier functions following renal ischemia/reperfusion (I/R) surgery. Notably, in NLRP3-knockout (NLRP3^−/−^) I/R mice, the deleterious effects of the HUA microbiota on renal injury and the intestinal barrier were eliminated.

**Conclusion:**

Our results demonstrate that HUA-induced gut dysbiosis contributes to the development of renal injury, possibly by promoting the production of gut-derived uremic toxins and subsequently activating the NLRP3 inflammasome. Our data suggest a potential therapeutic strategy for the treatment of renal diseases by targeting the gut microbiota and the NLRP3 inflammasome.

Video Abstract

**Graphical Abstract:**

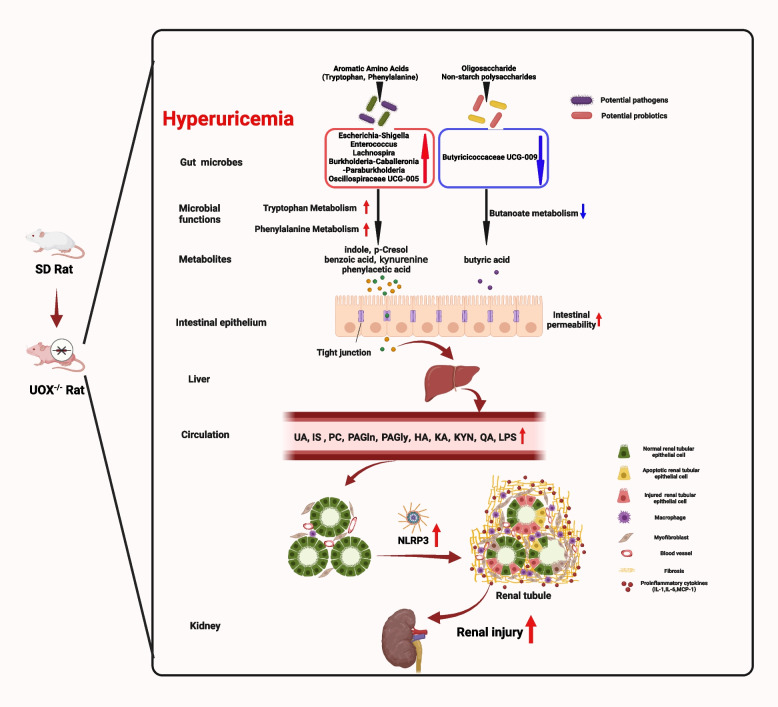

**Supplementary Information:**

The online version contains supplementary material available at 10.1186/s40168-024-01826-9.

## Background

The prevalence of hyperuricaemia (HUA) has continued to increase worldwide, with an evident reduction in the age of onset in recent years. A high prevalence of approximately 20% was seen between 2007 and 2016 in the United States [1]. In China, the prevalence of HUA has significantly increased from 11.1% in 2015–2016 to 14% in 2018–2019, reaching 32.3% in the 18–29-year-old men [2]. HUA is a metabolic disease caused by purine metabolic disorders, mainly due to the increased formation or reduced excretion of uric acid (UA). HUA is often associated with many diseases including gout [3], type 2 diabetes [4], cardiovascular diseases [5], and chronic kidney disease (CKD) [6]. Renal dysfunction is one of the most common complications of HUA. In addition, renal dysfunction also contributes to the elevation of serum UA levels since the kidneys are the major organs responsible for the excretion of UA. Epidemiological research has suggested that HUA is an independent risk factor of CKD [7]. Possible mechanisms of HUA-induced renal injury include renal tubular injury, inflammatory cell infiltration, and subsequent tubulointerstitial fibrosis [8, 9].

The gut microbiota is essential to maintain health. It is currently known that the gut microbiota is a critical player in the gut-kidney axis, playing a vital role in kidney health [10]. Gut dysbiosis has been implicated in various kidney disease, leading to the increased production of gut-derived uremic toxins such as indoxyl sulphate (IS), p-cresyl sulphate (PCS), and trimethylamine N-oxide (TMAO) [11–13]. While the kidneys are the main route of UA excretion, the intestinal tract also plays an important role in UA excretion. Approximately, 1/3 of UA is excreted into the intestinal tract and further metabolized by gut bacteria. Hence, it is reasonable to assume that the gut microbiota undergoes changes in response to alterations in UA metabolism. Several clinical and animal studies have demonstrated notable differences in the gut microbiota between individuals with HUA and gout when compared to healthy controls [14–17]. However, the specific role and underlying mechanism of gut dysbiosis induced by HUA in the development of renal injury still remain unclear.

Inflammation, an immune response to counteract harmful pathogens, serves as a major pathogenic mechanism in both CKD and acute kidney injury (AKI). In a damaged renal interstitium, the presence of inflammatory cells is commonly observed in failing kidneys and is inversely correlated with renal function [18]. The development and activation of the immune system heavily rely on interactions between the host and the gut microbiota, and an imbalance can result in sustained immune activation or inappropriate immune suppression [19]. Notably, nod-like receptor protein 3 (NLRP3), an inflammatory regulator, has been found to be activated in mouse models of CKD and AKI, as well as in human kidney diseases [20–22]. The NLRP3 inflammasome is a group of pattern recognition receptors involved in various innate immune responses to both microbial and nonmicrobial stimuli [23]. Previous research has indicated that the NLRP3 inflammasome is implicated in the regulation of intestinal homeostasis and can be activated by bacterial products such as lipopolysaccharide (LPS) and gut-derived uremic toxins like IS [24–26]. Therefore, it is reasonable to hypothesize that the NLRP3 inflammasome participates in the cross-talk between the gut microbiota, inflammation, and the kidney axis.

The susceptibility of humans to HUA can be attributed to the inactivation of the uricase gene (also known as uric acid oxidase, UOX) during primate evolution. UOX is expressed in the rodent liver, which degrades UA into allantoin, hindering the establishment of stable HUA models. The primary approach to create HUA models involves the administration of drugs that promote purine synthesis or inhibit UOX activity. However, these drug-induced models often exhibit unstable serum UA levels. Alternatively, a novel strategy has emerged, which involves genetically modifying UOX to establish stable HUA models. As anticipated, UOX-knockout mice develop HUA spontaneously. However, only a small number of these mice can survive to maturity due to the presence of severe renal injury [27].

In the current study, a UOX-knockout (UOX^−/−^) rat model on a Sprague-Dawley background was used in conjunction with a microbiome and metabolomics analyses to explore the impact of the gut microbiota on the progression of HUA-induced renal injury and its underlying mechanism. Through the utilization of this model, our research demonstrated that UOX^−/−^ rats exhibited notable HUA with renal injury, characterized by increased kidney fibrosis and inflammation. Additionally, there were obvious changes in the composition of the gut microbiota, as well as elevated levels of uremic toxins derived from bacteria. Further investigation through faecal microbiota transplantation (FMT) in both wild-type (WT) and NLRP3-knockout (NLRP3^−/−^) mice revealed that the microbiota associated with HUA played a vital role in the progression of renal injury by activating the NLRP3 inflammasome.

## Results

### UOX^−/−^ rats developed significant HUA and renal dysfunction

**Fig. 1:**
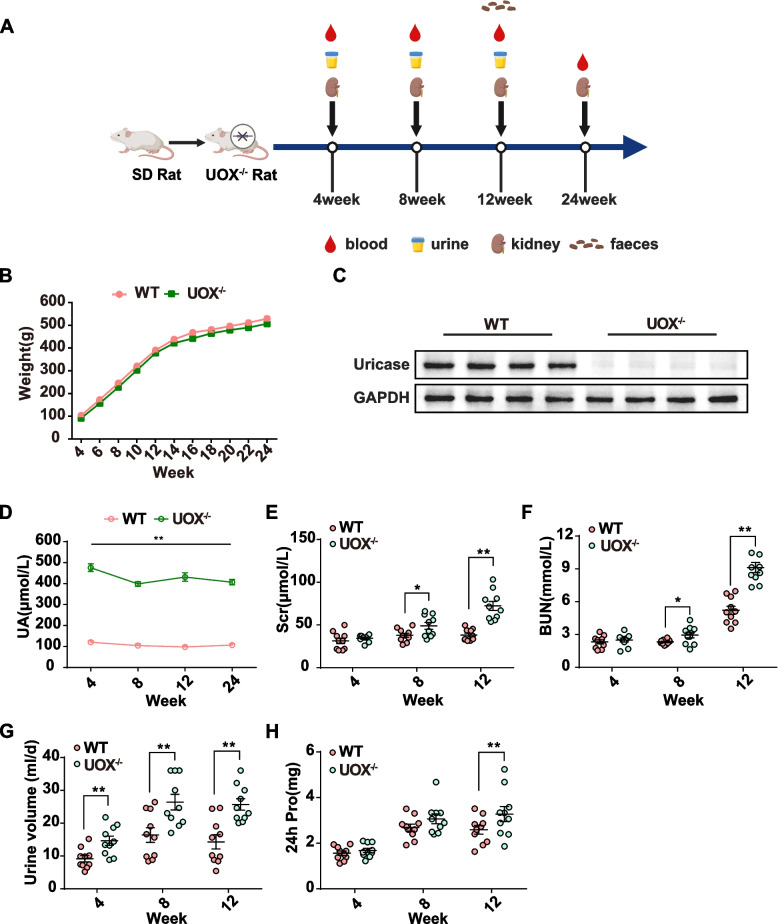
UOX^–/–^ rats developed significant HUA and renal dysfunction. **A** Timeline of the experiment (blood, urine, kidneys, and faeces were collected in different time points). **B** Body weight changes (*n* = 10). **C** Expression of hepatic UOX (*n* = 4). **D** Serum UA of UOX^–/–^ and WT rats from 4 to 24 weeks of age (*n* = 10). **E**–**H** Levels of SCR, BUN, 24-h urine volume, and urine protein in UOX^–/–^ and WT rats at 4, 8, and 12 weeks of age (*n* = 10). Data are represented as mean ± standard error of the mean (SEM). Statistical comparison was performed using one-way repeated measurement analysis of variances. *p < 0.05; **p < 0.01

Blood, urine, faeces, and kidneys were collected at different time points from the rat models (Fig. 1A). As shown in Fig. 1B, there was no significant difference in body weight between WT and UOX^−/−^ rats. The UOX protein dramatically reduced in the livers of UOX^−/−^ rats compared to that of WT rats as determined by Western blot (Fig. 1C). Consistently, UOX^−/−^ rats had significantly higher serum UA levels when compared to WT rats (Fig. 1D). Serum UA levels stabilized at about 400 μmol/L in the UOX^−/−^ rats from 4 to 24 weeks of age. No differences were found in food intake between the two groups, but the water intake in UOX^−/−^ rats was significantly increased when compared to the WT controls (Fig. S1A and B). A Kaplan-Meier analysis of survival rates over a 24-week observation period showed that there was no significant difference between the two groups (Fig. S1C). To investigate the effects of HUA on the kidney, renal function was determined in WT and UOX^−/−^ rats from 4 to 12 weeks of age. Compared to WT controls, serum creatinine (SCR) and blood urea nitrogen (BUN) were significantly elevated at 8 weeks, and 24-h urine proteins were increased at 12 weeks in UOX^−/−^ rats (Fig. 1E–H). Collectively, these findings indicate that the UOX^−/−^ rats were effectively established as a model for persistent and spontaneous HUA, and that this condition resulted in severe renal dysfunction.

### HUA causes significant renal histopathological injury

**Fig. 2:**
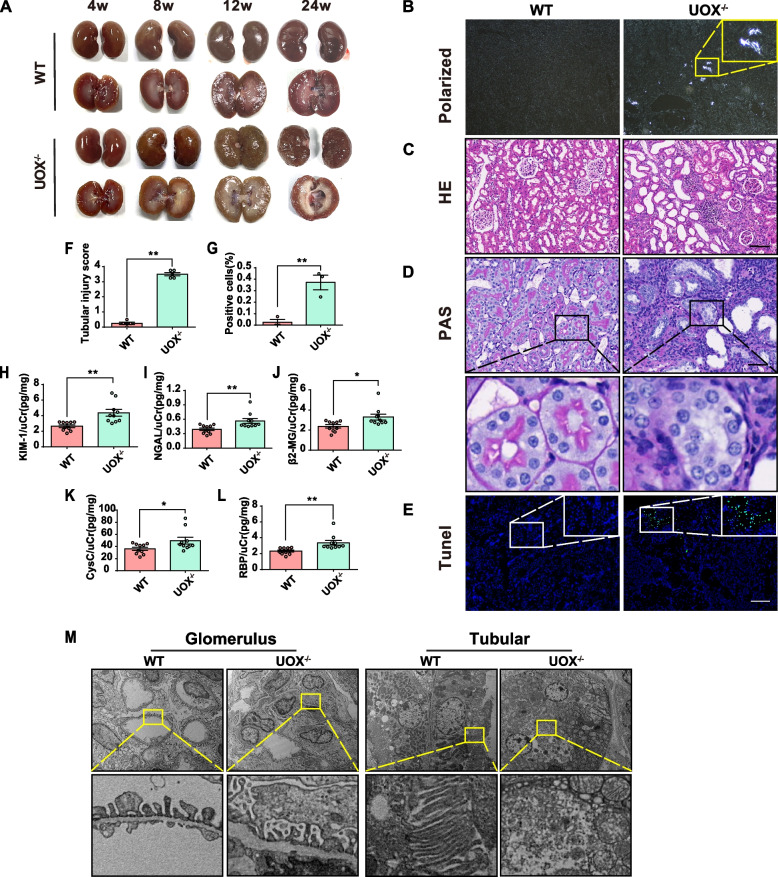
HUA causes significant renal histopathological injury. **A** Kidney morphology of UOX^–/–^ and WT rats at 4, 8, 12, and 24 weeks of age. **B** UA crystals detected under polarized light in the kidneys of rats at 12 weeks of age. **C** H&E staining of kidneys in rats at 12 weeks of age. Scale bar, 100 μm. **D** and **F** PAS staining of kidney in rats at 12 weeks of age and renal tubular injury scores (*n* = 5). Scale bar, 50 μm. **E** and **L** Tunnel staining of the kidney and quantification of positive staining cells (*n* = 3). Scare bar, 100 μm. **G**–**K** Urinary KIM-1, NGAL, RBP, β2-MG, and CysC levels, normalized by urinary creatinine, in rats at 12 weeks of age (*n* = 10). **M** TEM of kidneys in rats at 12 weeks of age. Data are represented as mean ± SEM. Statistical comparison was performed using two-tailed unpaired Student’s *t*-tests. *p < 0.05; **p < 0.01

Kidney gross morphology was examined from 4 to 24 weeks of age. Compared with WT controls, UOX^−/−^ rats exhibited significant impairment of kidney morphology as shown by a less smooth surface and the presence of structural damage (Fig. 2A). Kidney histopathology was examined at 12 weeks of age. Needle-like UA crystals were observed in kidney interstices of UOX^−/−^ rats under polarized light (Fig. 2B). Haematoxylin and eosin (H&E) staining showed renal tubule dilatation with disordered epithelial cell arrangement and inflammatory cell infiltration in the kidney of UOX^−/−^ rats (Fig. 2C). Pathologic morphology was then detected by periodic acid-Schiff (PAS) staining, a method that identifies injured tubules through the detection of glycogen content. PAS staining showed significant renal tubular injury in the kidney of UOX^−/−^ rats, characterized by tubular dilation and damaged brush border membrane, as well as detached epithelial cells in tubular lumens (Fig. 2D and F). Terminal deoxynucleotidyl transferase dUTP nick-end labelling (Tunel) staining revealed that HUA promotes cell apoptosis in the kidneys of UOX^−/−^ rats (Fig. 2E and L). Urinary N-acetyl-b-D-glucosaminidase (NAGL), kidney injury molecule 1 (KIM-1), retinol-binding protein (RBP), β2-microglobulin (β2-MG), cystatin C (CysC), and biomarkers of tubular damage were detected by ELISA kits. Consistent with the PAS staining, urinary NAGL, KIM-1, RBP, β2-MG, and CysC levels were significantly elevated in UOX^−/−^ rats (Fig. 2G–K). Furthermore, transmission electron microscopy (TEM) revealed the glomerular basement membrane thickening, mesangial cell proliferation, and tubular structural destruction in the kidney samples (Fig. 2M). These results suggest that HUA causes significant renal injury, and in particular tubular damage, in UOX^−/−^ rats.

### HUA promotes renal fibrosis and activation of the NLRP3 inflammasome in the kidney

**Fig. 3:**
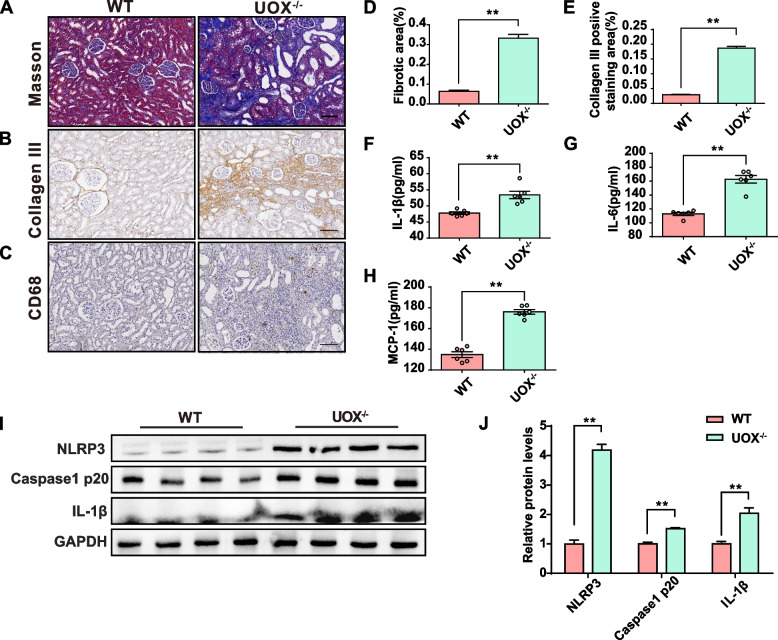
HUA promotes renal fibrosis and activation of the NLRP3 inflammasome in the kidney. **A** and **D** Masson trichrome staining and quantification of fibrotic areas (*n* = 3). **B** and **E** Collagen III staining in kidneys of rats (*n* = 3). Scale bar, 100 μm. **C** Immunostaining of CD68 in the kidneys of rats. Scale bar, 100 μm. **F**–**H** Inflammatory cytokine levels in the serum (MCP-1, IL-1β, IL-6) (*n* = 6). **I** and **J** Expression and quantification of NLRP3, Caspase1 p20, and IL-1β proteins in the kidneys of rats (*n* = 4). Data are represented as mean ± SEM. Statistical comparison was performed using two-tailed unpaired Student’s *t*-tests. *p < 0.05; **p < 0.01

Fibrosis is a common pathological feature of multiple kidney diseases, and chronic inflammation is considered a typical pathological characteristic of HUA. Masson trichrome and collagen III staining revealed apparent interstitial fibrosis in the kidneys of UOX^−/−^ rats (Fig. 3A, B, D, and E). Furthermore, immunofluorescence staining using CD68 revealed an elevated number of macrophages in the kidneys of HUA rats (Fig. 3C). Consistently, the expressions of serum inflammatory factors, including MCP-1, IL-1β, and IL-6, were up-regulated in UOX^−/−^ rats (Fig. 3F–H). Western blot results showed that the protein expressions of NLRP3, cleaved caspase1 p20, and IL-1β proteins were up-regulated (Fig. 3I and J), indicating that the NLRP3 inflammasome was activated in the kidneys of UOX^−/−^ rats when compared to WT rats. These results suggest that HUA activates the NLRP3 inflammasome and promotes kidney fibrosis in the UOX^−/−^ rats.

### HUA leads to gut microbiota dysbiosis and impaired intestinal barrier function

**Fig. 4:**
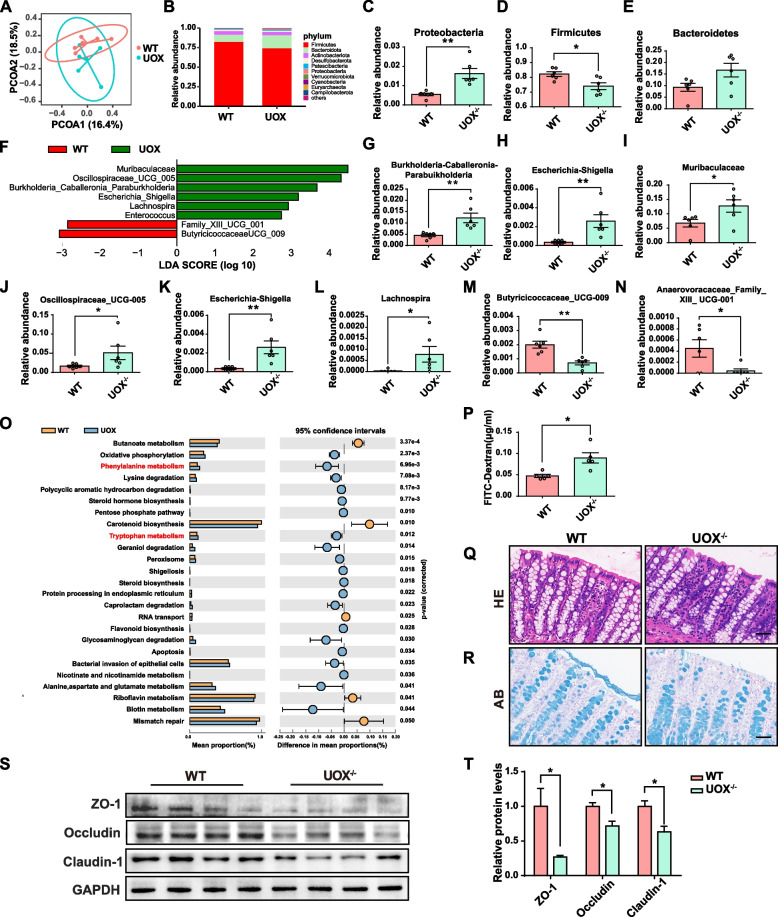
HUA leads to gut microbiota dysbiosis and impaired intestinal barrier function in UOX^−/−^ rats. **A** PCoA analysis using Bray-Curtis distances between WT and UOX^−/−^ rats (*n* = 6). **B**–**E** Relative abundance profiles at phylum level (*n* = 6). **F** Linear discriminant analysis (LDA) scores showing the most differentially abundant taxa significantly enriched in the gut microbiota of WT and UOX^−/−^ rats. **G**–**N** Relative abundance profiles at a genus level (*n* = 6). **O** Predicted KEGG functional pathway differences inferred from 16S rRNA gene sequences obtained using PICRUSt between WT and UOX^−/−^ rats. **P** The intestinal permeability of WT and UOX^−/−^ rats was detected by FITC-dextran (*n* = 5). **Q**–**R** H&E and AB staining of the colon of rats. Scale bar, 50 μm. **S**–**T** The expression of tight junction proteins in the colon of rats (*n* = 4). Data are represented as mean ± SEM. Statistical comparison was performed using Wilcox rank-sum tests or two-tailed unpaired Student’s *t*-tests. *p < 0.05; **p < 0.01

To investigate the effects of HUA on the gut microbiota, we performed 16S rRNA sequencing to reveal differences in the gut microbial community profiles between groups. Faecal samples were collected from UOX^−/−^ littermate rats and WT littermate control rats to reduce bias based on genetic backgrounds. Microbial richness and evenness were similar between WT and UOX^−/−^ rats based on *α*-diversity indexes, including ACE, Shannon, and Chao1 indexes (Fig. S2A–C). Principal co-ordinates analysis (PCoA) based on the Bray-Curtis distance showed a relative separation in microbial communities between groups (Fig. 4A). At the phylum level, the gut microbiota of both WT and UOX^−/−^ rats were mainly composed of Firmicutes, Bacteroidetes, Actinobacteriota, and Proteobacteria (Fig. 4B). Proteobacteria were significantly increased in UOX^−/−^ rats when compared to the WT rats, while Firmicutes were reduced (Fig. 4C and D). The identification of differentially abundant faecal bacterial taxa at the genus level between groups was performed using a linear discriminant analysis effect size (LEfSe) analysis, as shown in Fig. 4E. The LEfSe analysis results showed that six bacterial genera, including *Escherichia-Shigella*, were enriched in the UOX^−/−^ rats, while the other two taxa were enriched in the WT rats (Fig. 4G–N). Furthermore, using KEGG annotation and functional enrichment, 25 functional categories that exhibited different enrichment levels between the UOX^−/−^ and WT rats were identified (Fig. 4O). Notably, functions associated with gut-derived uremic toxins, including tryptophan and phenylalanine metabolisms, were increased in the UOX^−/−^ rats. In addition, the KEGG pathway analysis also revealed that a HUA-perturbed microbiota was associated with increased bacterial invasion of epithelial cells and decreased butanoate metabolism.

To assess whether a perturbed gut microbiota due to HUA had an impact on intestinal barrier function, intestinal permeability was assessed using FITC-dextran. The intestinal permeability of UOX^−/−^ rats was significantly increased when compared to the WT rats (Fig. 4P). In rats with HUA, the histological examination of colonic tissues using HE and alcian blue (AB) staining revealed several notable findings. These included a thinner mucus layer, loss of goblet cells, and increased infiltration of inflammatory cells (Fig. 4Q and R). Additionally, the expression levels of tight junction proteins, such as ZO-1, occludin, and claudin-1, in the colon of UOX^−/−^ rats were lower when compared to those in WT rats (Fig. 4S and T). Taken together, these results suggest that HUA in UOX^−/−^ rats contributes to gut microbiota dysbiosis and compromised intestinal barrier function.

### Gut-derived uremic toxins are significantly increased in the kidney of rats with HUA

**Fig. 5:**
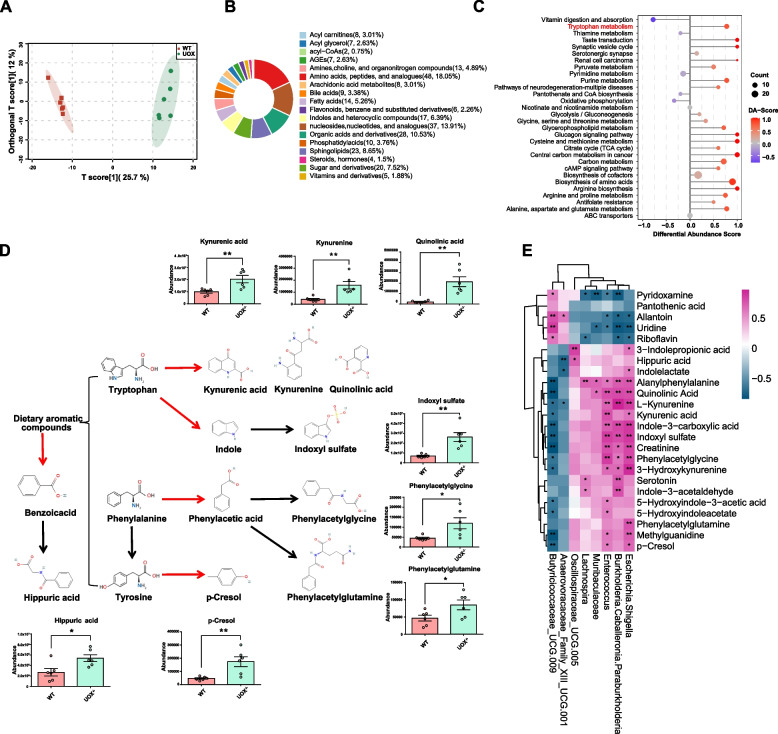
Gut-derived uremic toxins are significantly increased in the kidneys of rats with HUA. **A** OPLS-DA model discrimination based on metabolic profiles in kidney samples between WT and UOX^−/−^ groups (*n* = 6). **B** Classification of differential metabolites between WT and UOX^−/−^ rats. **C** Differential abundance score of the KEGG pathway between WT group and UOX^−/−^ group. **D** Differential metabolites involved in the metabolism of aromatic compounds and their relative intensity in WT and UOX^−/−^ rats (*n* = 6). Red arrows indicate processes mediated by gut microbes, while black arrows indicate processes mediated by host. **E** Pearson correlation between different genus and metabolites. Red indicates a positive correlation, while blue indicates a negative correlation. Data are represented as mean ± SEM. Statistical comparison was performed using two-tailed unpaired Student’s *t*-tests. *p < 0.05; **p < 0.01

Gut-derived metabolites can be critical mediators of the gut-kidney axis and thus play an important role in kidney health. The results of a KEGG function prediction analysis of the gut microbiome suggest that gut dysbiosis induced by HUA increases the production of gut-derived uremic toxins. Therefore, a targeted metabolomics analysis was used to compare the kidney metabolomic profiles between UOX^−/−^ and WT rats. The results of OPLS-DA model showed a clear separation between WT and UOX^−/−^ rats (Fig. 5A). A random permutation test with 200 permutations was further performed to evaluate the robustness of the OPLS-DA model (Fig. S3). Results indicated that the OPLS-DA model was not overfit and could be further processed.

With a VIP *>* 1 threshold and a *p <* 0.05, a total of 266 metabolites between the WT and UOX^−/−^ groups were identified (Fig. S4). Of these, 180 metabolites were up-regulated, and 86 metabolites were down-regulated in UOX^−/−^ rats when compared to the WT group. Results of a classification revealed that these metabolites mainly belonged to amino acids, peptides, and analogues, nucleosides, nucleotides, and analogues, organic acids and derivatives, sphingolipids, sugar and derivatives, indoles and heterocyclic compounds, among others (Fig. 5B). Among the altered metabolites, several uremic toxins, including IS, p-Cresol (PC), hippuric acid (HA), phenylacetylglutamine (PAGln), phenylacetylglycine (PAGly), kynurenic acid (KA), L-kynurenine (KYN), quinolinic acid (QA), and methylguanidine (MG), were up-regulated in UOX^−/−^ rats (Fig. 5D). Most of these toxins are known to be derived from the metabolism of dietary aromatic compounds including tryptophan, phenylalanine and tyrosine by the gut microbiota.

The altered metabolites were further analysed using KEGG pathways (Fig. 5C). Consistent with the gut microbiome, the metabolite-enriched KEGG pathway analysis showed that the tryptophan metabolism was up-regulated in UOX^−/−^ rats. In addition, biosynthesis of amino acids, purine metabolism, pyruvate metabolism, cysteine and methionine metabolism, TCA cycle, and cAMP signalling were also up-regulated, while vitamin digestion and absorption, thiamine metabolism, and pyrimidine metabolism were down-regulated in UOX^−/−^ rats. Pearson correlation analysis was further performed to understand the association between differentially enriched microbes and metabolites (Fig. 5E). The correlation analysis showed that *Escherichia-Shigell*a, *Burkholderia*, *Caballeronia*, *Paraburkholderia*, and *Enterococcus* enriched in UOX^−/−^ group had a strong positive correlation with gut-derived uremic toxins, while Butyricicoccaceae *UCG-009* enriched in WT group had a negative correlation with these altered metabolites. Moreover, correlation analysis between metabolites and renal function parameters revealed that these gut-derived uremic toxins had a strong positive correlation with UA levels and renal function parameters (Fig. S5). Correlations among discriminative microbes, differential metabolites, and renal function parameters are also shown in the network (Fig. S6).

These results indicated that gut-derived uremic toxins up-regulated in UOX^−/−^ rats may be involved in HUA-induced renal injury.

### Transplantation of the “HUA microbiota” promotes renal injury and the activation of NLRP3 in ischemia/reperfusion (I/R) mice

**Fig. 6:**
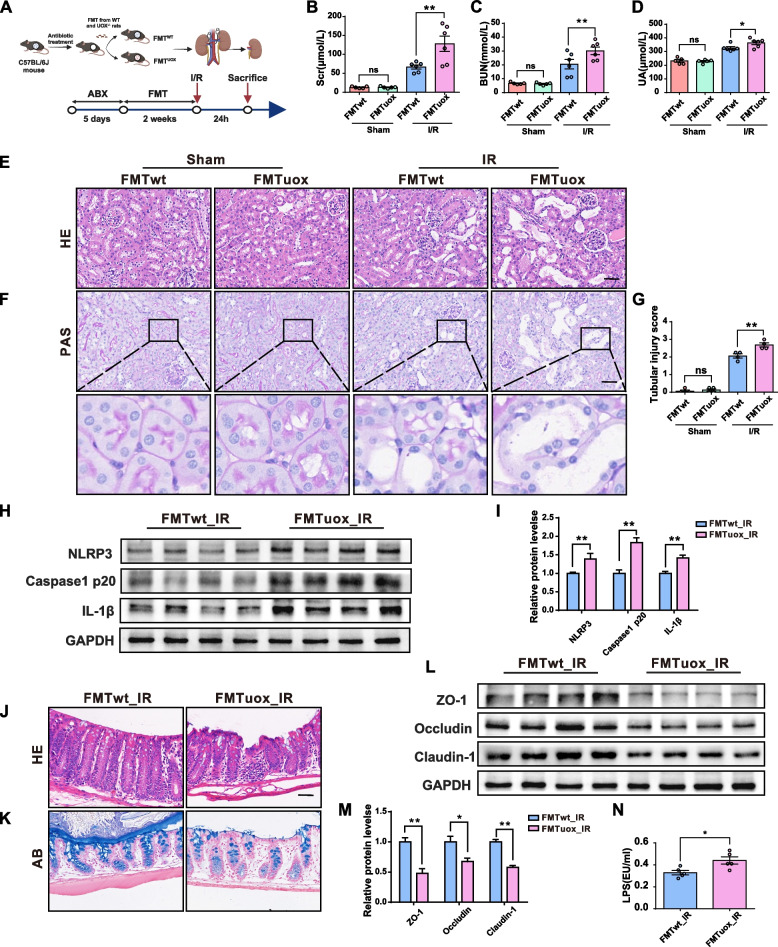
Transplantation of “HUA microbiota” promotes renal injury and the activation of NLRP3 in I/R mice. **A** The schedule of I/R after FMT from WT or UOX^−/−^ rats to recipient mice. **B**–**D** Levels of SCR, BUN, and UA in recipient mice (*n* = 6). **E**–**F** H&E and PAS staining of kidneys in recipient mice. Scale bar, 50 μm. **G** Renal tubular injury scores (*n* = 4). **H**–**I** Expression and quantification of NLRP3, Caspase1 p20, and IL-1β expressions in the kidneys of rats (*n* = 4). **J**–**K** H&E and AB staining of colons in recipient mice. Scale bar, 50 μm. **L**–**M** Expression and quantification of tight junction proteins (ZO-1, occludin, claudin-1) in the colon of mice (*n* = 4). **N** Levels of serum LPS in recipient mice after I/R surgery (*n* = 6). Data are represented as mean ± SEM. Statistical comparison was performed using one-way analysis of variance or two-tailed unpaired Student’s *t*-tests. *p < 0.05; **p < 0.01; *ns* means no significant difference

To further confirm the contribution of the gut microbiota to HUA-induced renal injury, faecal microbiota was transplanted from WT littermate rats and UOX^−/−^ littermate rats into gut microbiota-depleted mice which previously underwent 5 days of combined ampicillin, neomycin, vancomycin, and metronidazole antibiotic (ABX) treatment. The depletion of microbiota in ABX-treated mice was evidenced by a significant decrease in the bacterial DNA load and absent culturable bacteria in blood agar plating (Fig. S7). I/R injury was performed after 2 weeks of FMT to induce acute renal injury (Fig. 6A). Cecum contents from the recipient mice were collected for 16S rRNA gene sequencing to investigate the effects of FMT on the gut microbiota in sham or I/R mice (Fig. S8). PCoA analysis based on the relative abundance of genus showed a relative separation in microbial communities between mice recolonized with HUA and control microbiota. Consistent with the above findings in rats, the predicted KEGG functional pathway analysis showed that tryptophan metabolism was significantly increased in both sham and I/R mice recolonized with HUA microbiota (Fig. S8F-G).

Further evidence for the increased tryptophan metabolism was shown by the increased level of serum IS in HUA microbiota recipient mice following I/R injury (Fig. S8H). Moreover, the function associated with NOD-like receptor signalling pathway was increased in HUA microbiota recipient mice after I/R injury (Fig. S8G). These results indicated that the gut microbiota of UOX^−/−^ and WT rats were successfully reestablished in recipient mice through FMT.

The effects of the HUA microbiota were then investigated on renal injuries of mice after FMT. As shown in Fig. 6B–C and E–G, the HUA microbiota did not directly induce renal injury. In contrast, mice receiving the HUA microbiota exhibited more severe renal injury following the I/R surgery when compared to the control group. This was evidenced by elevated SCR and BUN levels, along with enhanced renal tubular injury. Consistently, the UA levels in HUA microbiota recipient mice were higher than that in controls after I/R injury, while no difference was seen in sham mice (Fig. 6D). Subsequently, the activity of the NLRP3 inflammasome, one of the NOD-like receptors, was assessed after I/R surgery in the kidneys of HUA microbiota recipient mice. As expected, the expressions of NLRP3, cleaved caspase-1 p20, and IL-1β were significantly up-regulated in I/R mice recolonized with HUA microbiota (Fig. 6H and I).

The effects of the HUA microbiota on the intestinal barrier of recipient mice were further investigated. H&E and AB staining of colonic sections showed a thinner mucus layer and increased inflammatory cell infiltrates in the HUA microbiota recipient mice when compared to controls (Fig. 6J and K). The impaired intestinal barrier of HUA microbiota recipient mice was also indicated by increased levels of serum LPS (Fig. 6N) and decreased expression of tight junction proteins including ZO-1, occludin, and claudin-1 (Fig. 6L and M). Collectively, these findings unveil the role of the HUA microbiota in promoting renal injury, triggering activation of the renal NLRP3 inflammasome and compromising the integrity of the intestinal barrier.

### Activation of NLRP3 is responsible for the harmful effect of the “HUA microbiota” on renal injury

**Fig. 7:**
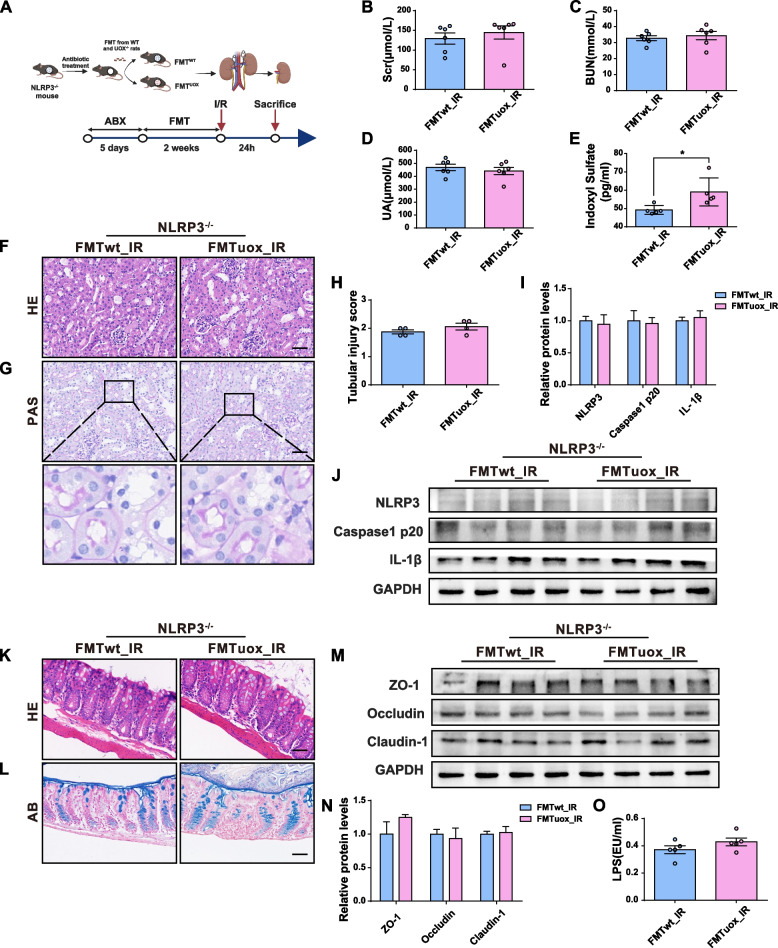
Activation of NLRP3 is responsible for the harmful effect of the “HUA microbiota” on renal injury. **A** The schedule of I/R after faecal FMT from WT or UOX^−/−^ rats to recipient NLRP3^−/−^ mice. **B**–**E** Levels of SCR, BUN, UA, and IS in recipient mice (*n* = 6). **F**–**G** H&E and PAS staining of kidneys in recipient mice. Scale bar, 50 μm. **H** Renal tubular injury scores (*n* = 4). **I**–**J** Expression and quantification of NLRP3, Caspase1 p20, and IL-1β expressions in the kidneys of rats (*n* = 4). **K**–**L** HE and AB staining of colons in recipient mice. **M**–**N** Expression and quantification of tight junction proteins (ZO-1, occludin, claudin-1) in the colon of mice (*n* = 4). **O** Levels of serum LPS in recipient mice after I/R. Data are represented as mean ± SEM. Statistical comparison was performed using two-tailed unpaired Student’s *t*-tests. *p < 0.05

The predicted KEGG functional pathway analysis indicated that the NOD-like receptor signalling pathway may be involved in HUA microbiota-related renal injury. To investigate the potential involvement of the NLRP3 inflammasome in mediating the promotion effects of HUA microbiota on renal injury, an additional experiment involving I/R surgery in NLRP3^−/−^ mice after microbiota transplantation was conducted (Fig. 7A). In contrast to WT mice, no significant differences were observed in SCR, BUN, UA levels, and histological changes in the kidneys of NLRP3^−/−^ mice transplanted with different microbiota following I/R surgery, although higher levels of gut-derived uremic toxin IS were still detected in the HUA microbiota recipient mice (Fig. 7B-H). As expected, the expression of NLRP3, cleaved caspase-1 p20, and IL-1β in the kidneys of NLRP3^−/−^ mice after I/R surgery remained similar (Fig. 7I and J). Furthermore, the intestinal barrier impairment induced by the HUA microbiota in WT mice was not observed in NLRP3^−/−^ mice, as evidenced by H&E and AB staining, expression of tight junction proteins, and serum LPS levels (Fig. 7K–O). These findings suggest that activation of the NLRP3 inflammasome by the HUA microbiota is responsible for the exacerbation of renal injury and intestinal barrier impairment in I/R mice.

## Discussion

In the present study, in addition to evident renal injury, we found that HUA led to gut microbiota dysbiosis and compromised intestinal barrier function in a UOX^−/−^ rat model. Gut dysbiosis in these rats was accompanied by marked alteration of the kidney metabolome and in particular the accumulation of gut derived uremic toxins related to dietary aromatic amino acids metabolism. FMT experiments further confirmed that the HUA microbiota promoted renal injury, and that activation of the NLRP3 inflammasome may mediate this process.

Serum UA levels are often unstable and can fluctuate widely in animal models of drug-induced HUA due to the expression of UOX in the liver. As such, a HUA mouse model was established by knocking out the UOX gene. However, few of these mice could survive to maturity. Therefore, a UOX^−/−^ rat model was used in this study. The sustained high levels of UA and normal survival rate indicated that UOX^−/−^ rats were a useful model for studying HUA and related gut microbiota dysbiosis. Remarkably, HUA also caused activation of the NLRP3 inflammasome in the kidney of UOX^−/−^ rats.

Increasing evidence has demonstrated that gut microbiota and microbiota-derived metabolites are critical mediators of gut-kidney axis, playing a vital role in human health [28]. Gut microbiota dysbiosis has been implicated in multiple kidney diseases and can result in the increased production of microbiota-derived uremic toxins [29]. In addition, studies have shown that gut microbiota dysbiosis occurs in HUA patients and murine models of HUA [17, 30, 31]. However, whether microbiota dysbiosis induced by HUA promotes the progression of renal injury and how this occurs mechanistically remains unclear. In this study, HUA did not cause significant changes of gut microbiota diversity in UOX^−/−^ rats. We hypothesized that alterations in the composition of the gut microbiota induced by HUA, rather than changes in the microbiota diversity itself, may play a role in the progression of renal injury. The gut microbiome in both WT and UOX^−/−^ rats was dominated by Firmicutes, Bacteroidetes, Proteobacteria, and Actinobacteria, which are typical gut microbiome structures in rats. Proteobacteria, a major phylum of gram-negative bacteria, including a wide variety of pathogens and opportunistic pathogens, has been found to be increased in CKD and HUA rats [32, 33]. In line with this evidence, we found that Proteobacteria was enriched in UOX^−/−^ rats. At genus level, we detected an enrichment of *Escherichia-Shigella*, *Enterococcus*, *Lachnospira*, and Oscillospiraceae *UCG-005* and the depletion of Butyricicoccaceae *UCG-009* in the UOX^−/−^ rats. A correlation analysis showed that these enriched genus in UOX^−/−^ rats were positively correlated with uremic toxins, while the depleted Butyricicoccaceae *UCG-009* negatively correlated with uremic toxins. The results from our functional prediction analysis revealed that uremic toxin synthesis was activated in the microbiome of UOX^−/−^ rats, particularly in relation to tryptophan and phenylalanine metabolism.

*Escherichia-Shigella* and *Enterococcus* are both opportunistic pathogens responsible for a wide variety of intestinal and extraintestinal infections in animals and humans. A previous study showed that *Escherichia-Shigella* is not only increased in CKD patients but is also a diagnostic biomarker in these patients [34]. *Escherichia-Shigella* and *Escherichia coli* are both members of the Enterobacteriaceae family. The *Escherichia coli* genome contains genes encoding tryptophanase capable of metabolizing dietary tryptophan to indole and producing gut-derived uremic toxins such as IS, indicating the potential effect of *Escherichia-Shigella* on uremic toxin production in the rats [35]. In addition, it has been reported that a high sulphur amino acid-containing diet results in post-translationally modified microbial tryptophanase activity, thus reducing uremic toxin-producing activity and ameliorating progression to CKD in mice [36]. The rise in *Lachnospira* has previously been shown to associate with the levels of TMAO, an important toxin that promotes the progression of CKD and increases the risk of cardiovascular events [37–39]. *Oscillospira*, one of the species assigned to Oscillospiraceae, has been linked with inflammatory indices in CKD patients [40]. Moreover, depletion of Butyricicoccaceae *UCG-009* genera observed in the UOX^−/−^ rats is consistent with previously reported findings in UOX^−/−^ mice [30]. Microbial alterations in the gut may affect the permeability of the intestinal mucosal barrier and lead to translocation of bacteria and bacteria-derived uremic toxins into systemic circulation. In line with these findings, we observed an elevated permeability of the intestinal mucosal barrier in the UOX^−/−^ rat model.

Changes of microbiota composition and functional prediction analysis suggested that gut microbiota metabolites may be involved in HUA-induced renal injury. As expected, several gut-derived uremic toxins including IS, PC, HA, PAGln, PAGly, KA, KYN, and QA were increased in the kidneys of UOX^−/−^ rats. All of these microbiota-dependent metabolites are known to accumulate in CKD and contribute to pathogenesis and disease progression by inducing renal injury, inflammation, and fibrosis [12]. Tryptophan is an essential amino acid for humans, which cannot be synthesized and must be obtained from diet. A variety of uremic toxins result from the tryptophan metabolism. IS is a small protein-bound uremic toxin produced by the dietary tryptophan metabolism. Tryptophanase of bacteria such as *Escherichia coli* converts tryptophan to indole, which is absorbed into the host’s circulation and further sulphated into IS in the liver. IS has been implicated in CKD and may contribute to vascular and renal disease progression [29]. Furthermore, it was reported that genetic manipulation of *Bacteroides* sp. tryptophanase could modulate levels of IS and improve renal function in a gnotobiotic mouse model, suggesting a possible strategy for treatment of the renal disease by targeting the gut microbiota [41]. In both in vivo and in vitro studies, IS has demonstrated its nephrotoxic effects primarily by activating oxidative stress, inducing inflammation, and promoting fibrosis [42–44]. Additionally, studies have shown that IS can induce intestinal epithelial cell damage by inhibiting mitochondrial autophagy and decrease the expression of intestinal barrier proteins such as ZO-1, occludin, and Claudin-1 to impair the intestinal mucosal barrier [45]. KA, KYN, and QA are also uremic toxins that result from the kynurenine pathway of tryptophan metabolism, which can initiate inflammatory responses and mitochondrial damage [46]. In addition to tryptophan, the microbial metabolism of phenylalanine and tyrosine by the gut microbiota results in several compounds associated with kidney disease. PC is produced from phenylalanine and tyrosine catabolism by intestinal anaerobic bacteria. Serum PC levels were predictive of CKD progression and mortality [47, 48]. PC is mostly conjugated to PCS in enterocytes. Similar to IS, PCS also induces oxidative stress, inflammation, and renal fibrosis [49–51]. However, in our study, we found significantly increased levels of PC instead of PCS in UOX^−/−^ rat. Indeed, PC is a small phenolic compound with lipophilic properties. The main difference in molecular structure between PCS and PC is the replacement of hydroxyl with sulphate, which makes PCS a hydrophilic compound. As a result, it appears that PC is more capable of permeating the cell membrane and disrupting its functionality, leading to the production of biological toxicity, compared to PCS [52]. PAGln and PAGly are colonic microbial metabolites derived from phenylalanine fermentation. Microbes metabolize phenylalanine to phenylacetic acid, which undergoes glutamine (preferred in humans) or glycine (preferred in rodents) conjugation to form PAGln or PAGly. PAGln has been associated with cardiovascular disease and identified as an independent risk factor for major adverse cardiovascular events (myocardial infarction, stroke, or death) [53, 54]. It has been reported that PAGln/PAGly contribute to platelet activation and enhanced thrombosis potential via multiple adrenergic receptors [53]. In addition, HA was generated from dietary polyphenols by the gut microbiota. The gut microbiome converts dietary polyphenols into benzoic acid and further conjugates with glycine to form HA in the liver. A study suggested that HA contributed to the progression of renal fibrosis by disrupting redox homeostasis [55]. In general, all of these gut-derived metabolites are known to accumulate in CKD and contribute to pathogenesis and disease progression by inducing renal injury, inflammation, and fibrosis. Consistently, the results of a Pearson correlation analysis revealed a significantly positive correlation between these gut-derived uremic toxins and renal injury, as well as the levels of UA. Since these protein-bound uremic toxins are not efficiently removed by haemodialysis, modulation of the intestinal microbiota may contribute to the reduction of uremic toxins.

To further investigate the contribution of gut microbiota dysbiosis in the progression of HUA-induced renal injury, FMT was performed. Mice were selected as recipient animals due to their high degree of similarity with human genomes, physiology, and the availability of gene-edited mice. Since different floras compete for survival in the gut, treatment with one or more specific bacteria may lead to a relatively low survival rate, and that may not fully explain the effects of a microbiota perturbed by HUA. Therefore, we performed gut recolonization using whole faecal microbiota in this study. The recipient mice and rats were pretreated with a mixture of unabsorbable antibiotics for 5 days to kill all prior bacteria. Mice in all groups were transplanted with the corresponding gut microbiota to exclude possible bias associated with microbiota depletion and the FMT process itself. The recipient mice were treated with FMT in the first 3 days to rebuild gut microbiota composition and twice a week to reinforce microbiota transplantation. Given that HUA microbiota might not directly induce renal injury, classic I/R mice were chosen to investigate the effects of the HUA microbiota on the development of renal injury. As expected, recipient mice recolonized with the HUA microbiota did not show obvious signs of renal injury. However, these mice displayed more serious renal injury after I/R. In line with the findings of renal injury, there was an increase in gut-derived IS in recipient mice with the HUA microbiota following I/R. Interestingly, mice recolonized with the HUA microbiota had a higher UA level than controls after I/R, while no difference was seen in sham mice recolonized with different microbiota. Based on our observations, we concluded that the increase in UA levels may be attributed to the exacerbation of renal injury rather than the direct effects of the HUA microbiota. This is supported by the lack of differences found in sham mice that were recolonized with different microbiota. These findings suggest that gut microbiota dysbiosis induced by HUA may function as a contributor, rather than an initiator, in the renal injury of UOX^−/−^ rats. Therefore, although restoring the gut microbiota in HUA rats may not completely eliminate renal injury itself, it may potentially reduce secondary damage caused by gut-derived uremic toxins from the gut to the kidneys. This also suggests that UA-lowering therapy in combination with therapy targeting intestinal microbiota to correct gut dysbiosis may be an effective strategy for the treatment of HUA-induced renal injury.

In addition, we also found that colonization with the HUA microbiota impairs intestinal barrier integrity as indicated by altered mucus layers and reduced expression of tight junction proteins. Increased gut permeability was further confirmed by increased serum LPS levels. The results of this study illustrate that the presence of the HUA microbiota leads to compromised intestinal barrier integrity. This impairment allows the passage of bacterial products, such as LPS, as well as gut-derived uremic toxins, such as IS. Consequently, these substances activate an inflammatory response in the kidneys. Renal inflammation is vital to the development of both AKI and CKD, as well as the transition of AKI to CKD. The predicted KEGG functional pathway analysis indicated that the NOD-like receptor signalling pathway was increased in HUA microbiota recipient mice after I/R. Activation of the NLRP3 inflammasome has been implicated in multiple mouse models and human kidney diseases, including AKI and CKD. In both HUA and the FMT experimental models, we found that the HUA microbiota led to the activation of NLRP3 in the kidneys. LPS may function as a pathogen-associated molecular pattern (PAMP) to activate the NLRP3 inflammasome. Furthermore, IS and a mixture of several uremic toxins, including HA, KA, and KYN, has been reported to promote the activation of NLRP3 in kidney tubule cells [25]. It is tempting to speculate that the NLRP3 inflammasome may mediate the deleterious effects of the HUA microbiota. In NLRP3^−/−^ mice, although the presence of the HUA microbiota resulted in elevated serum IS levels, there were no significant differences observed in renal injury or intestinal barrier integrity between the two groups of mice that were recolonized with different microbiota. This lack of difference can be attributed to the absence of NLRP3, indicating that the NLRP3 inflammasome activation plays a crucial role in mediating the harmful effects of HUA microbiota on renal injury. Therefore, inhibition of activity of NLRP3 may also be beneficial in preventing the progression of HUA-induced renal injury.

## Conclusion

In summary, our findings provide evidence that the dysbiosis of gut microbiota induced by HUA plays a significant role in the development of renal injury. Furthermore, we have identified NLRP3 activation as a potential mediator of this process. These results suggest that targeting the microbiota and NLRP3 inflammasome could be a promising strategy for the treatment of renal diseases.

## Methods

### Animals

UOX^−/−^ rats were obtained by intercrossing UOX^+/−^ rats (Nanjing Biomedical Research Institute of Nanjing University, China). Six-week-old C57BL/6J mice were obtained from the Guangdong Medical Laboratory Animal Center (Guangzhou, China). NLRP3^−/−^ mice were purchased from Cyagen Biosciences (Suzhou, China). To reduce bias based on genetic background, littermate controls were used for all experiments with genetically altered lines. Animals were kept under an automated 12-h light-dark cycle at a controlled temperature of 22 ± 2 °C, relative humidity of 50–60%, and had ad libitum access to a standard dry diet and tap water. The study was approved by the Standards for Animal Ethics in the Southern Medical University and performed in accordance with the relevant experimental animal guidelines and regulations.

### Serum UA and renal function parameters measurement

Serum UA was determined by a commercial kit (BioAssay Systems, CA, USA). A urine sample from each rat was collected over 24 h in a metabolic cage. SCR, BUN, and urine protein levels were determined using the respective analysis kits (Nanjing Jiancheng Bioengineering Institute, Nanjing, China). Urinary NAGL, KIM-1, RBP, and β2-MG levels were measured using respective rat ELISA quantitation kits, according to the manufacturer’s protocol (Boshen, Nanjing, China).

### Measurement of serum cytokines, LPS, and IS

ELISA kits were used to measure MCP1, IL-6, IL-1β (Boshen, Nanjing, China), LPS (Thermo Scientific, MA, USA,) and IS (Mlbio, Shanghai, China) in serum according to the manufacturer’s instructions.

### Histology and immunohistochemistry

Kidneys and colons were embedded in paraffin and sliced into 4-μm-thick sections by a routine procedure. H&E, PAS, Masson trichrome, and AB staining were conducted using standard protocols. Images were captured using a microscope and quantifies using the ImageJ software. Tubular injury (denudation of tubular cells, loss of brush border, flattening of tubular cells, formation of intratubular casts) was scored from 0 to 4 (0, no changes; 1, changes affecting < 25%; 2, changes affecting 25 to 50%; 3, changes affecting 50 to 75%; 4, changes affecting 75 to 100% of the section). Kidney sections were also obtained from absolute ethanol-fixed kidneys to detect uric acid crystals under polarized light.

Immunohistochemical detection of inflammation was performed using primary antibodies against macrophage marker CD68 (1:200, GB113109, Servicebio). Renal fibrosis was detected by collagen III (1:200, GB111629, Servicebio) staining.

## TEM

Kidneys were fixed with 2.5% glutaraldehyde and further fixed in PBS containing 1% osmium tetroxide. After being embedded, sectioned, and double-stained with uranyl acetate and lead citrate, images were captured using a transmission electron microscope (Hitachi, Japan).

### Western blot analysis

Kidney, liver, and colon tissues were homogenized in ice-cold RIPA lysis buffer, supplemented with protease and phosphatase inhibitor cocktail. The total protein concentration in supernatant was determined using a BCA protein assay kit. Equal amounts of protein samples were separated on SDS-PAGE gels and then transferred onto a PVDF membrane. The membranes were blocked with 5% non-fat milk in 0.1% TBST for 2 h at room temperature and then incubated with a primary antibody overnight at 4 °C. After washing three times with TBST, the membranes were incubated with the appropriate HRP-conjugated secondary antibody at room temperature for 2 h. Finally, the protein bands were detected with an ECL reagent and visualized using the ChemiDoc MP System (Bio-Rad, USA). The following primary antibodies were used: anti-GAPDH (ab9485, Abcam), anti-UOX (sc-166214, Santa Cruz), anti-NLRP3 (bs-6655R, Bioss), anti-caspase1 p20 (AF4005, Affinity), anti-IL-1β (bs-6319R, Bioss), anti-E-cadherin (14472S, CST), anti-N-cadherin (14215S, CST), anti-collagen IV (AF0510, Affinity), anti-α-SMA (50513S, CST), anti-ZO-1 (GB111402-100, Servicebio), anti-occludin (GB111401-100, Servicebio), and anti-claudin-1 (37-4900, Invitrogen). The relative density of protein bands was analysed using the ImageJ software.

### Metabolomic analysis

Kidneys of rats were prepared and deproteinized using methanol. The samples were then analysed using liquid chromatography-mass spectrometry by the BioProfile biotechnology company (Shanghai, China). Namely, this include a Shimadzu Nexera X2 LC-30AD system equipped with an ACQUITY UPLC HSS T3 column (1.8 μm, 2.1 × 50 mm Column, Waters) and a triple quadruple mass spectrometer (5500 QTRAP, AB SCIEX). Metabolites were detected in electrospray negative-ionization and positive-ionization modes. The mass spectrometer conditions were set as follows: negative-ionization: source temperature 550 °C, Ion Source Gas1 (GAS1): 40, Ion Source Gas2 (GAS2): 50, curtain gas (CUR): 35, and IonSpray Voltage Floating (ISVF): −4500 V and positive ionization: source temperature 550 °C, Ion Source Gas1 (GAS1): 40, Ion Source Gas2 (GAS2): 50, curtain gas (CUR): 35, and Ion Spray Voltage Floating (ISVF): 5500 V. Transitions were detected using the MRM mode.

The MultiQuant 3.0.2 software was used to extract the original MRM data of MT1000 KIT metabolites and obtain the peak area of each metabolite. The discriminating metabolites were obtained using a statistically significant threshold of variable influence on projection (VIP) values obtained from the OPLS-DA model and two-tailed Student’s *t*-test (*p*-value) on the normalized raw data. Metabolites with VIP greater than 1 and *p*-value less than 0.05 were considered statistically significant metabolites. The identified differential metabolites were used to perform cluster analyses with an R package. The enriched pathway analysis of changed metabolites was performed using the KEGG database.

### 16S rRNA gene sequencing and data analysis

Faecal samples from littermate rats and mice were collected under sterile conditions and stored at −80 °C before being assayed. Total genomic DNA was isolated from faecal samples using the QIAamp DNA Isolation Kit (Qiagen, Germany) according to manufacturer’s protocol. DNA concentration was determined by NanoDrop (Thermo Fisher Scientific, USA). DNA purity and integrity were evaluated using 1% agarose gel electrophoresis. Diluted DNA (1.0 ng/mL) was then used to amplify the V4 hypervariable region of the 16S rRNA gene with barcoded primers (515F, 5′-GTGCCAGCMGCCGCGGTAA-3′, 806R, 5′-GGACTACHVGGGTWTCTAAT-3′) and High-Fidelity PCR Master Mix with GC Buffer (New England Biolabs, USA). Thermocycling conditions were as follows: 98 °C pre-degeneration for 1 min, denaturation at 98 °C for 10 s, annealing at 50 °C for 30 s, and extension at 72 °C for 30 s, with a total of 30 cycles, followed by a final elongation step at 72 °C for 5 min. PCR products were subjected to 2.0% agarose gel electrophoresis, recovered, and purified using the TIANgel Purification Kit (Tiangen Biotech, China) and then pooled into equal concentrations. The purified product was used to prepare the Illumina DNA library. Sequencing libraries were generated using the TIANSeq Fast DNA Library Prep Kit (illumina) (Tiangen Biotech, China). The library quality was assessed on the Qubit@ 2.0 Fluorometer (Thermo Fisher Scientific, USA) and Agilent Bioanalyzer 2100 system. Finally, the library was sequenced on the Illumina platform using the 2 × 250 bp paired-end protocol. Sequencing data has been deposited in the Sequence Read Archive (SRA) of the National Center for Biotechnology Information (NCBI) (Bioproject: *PRJNA1026121*) to be released upon publication.

Raw data was analysed using the QIIME2 platform. Sequences were then quality filtered, denoised, merged, and chimera removed using the DADA2 plugin. Subsequently, the SILVA reference database classifier (version 138) for the classification of ASVs with a threshold of 97% sequence similarity. Determination of alpha- and beta-diversities were also conducted in QIIME2. The alpha-diversity indices, including ACE, Chao1, and Shannon index, were calculated on the data at an ASV level. The beta-diversity analysis was performed via PCoA using Bray-Curtis distances and displayed with the R software. Differentially abundant genera between groups were identified using linear discriminant analysis (LDA) and effect size (LEfSe) analysis. Only bacterial taxa reaching the LDA threshold of 2.0 were shown. PICRUSt based on OTUs was employed to predict the abundances of functional categories using KEGG orthologs (KO).

### Intestinal permeability assay

Intestinal permeability assays were performed according to a modified method described previously [56]. In short, rats were fasted for 4 h and anaesthetized with pentobarbital. A 10-cm segment of the ileum was separated beginning 3-cm proximal to the cecum, with well-protected superior mesenteric vessels. The proximal end of the isolated ileum was ligated with a 2.0 silk suture. A volume of 0.5-ml PBS containing 10 mg of FITC-dextran 4 kDa (FD-4; Sigma, USA) was injected into the lumen, after which the laparotomy was closed. After 30 min, a 1–2 ml blood sample was harvested via the portal vein. The blood was centrifuged at 4 °C, 3000 g for 10 min, and the serum was analysed for FD-4 concentration using a fluorescence microplate reader (BioTek, USA) at an excitation wavelength of 480 nm and an emission wavelength of 520 nm. Standard curves for calculating FITC-dextran concentrations in samples were obtained by diluting various amounts of FITC-dextran in PBS.

### FMT

FMT was performed according to a modified method described previously [57]. Briefly, 6-week-old male C57BL/6J mice received vancomycin (100 mg/kg), neomycin sulphate (200 mg/kg), metronidazole (200 mg/kg), and ampicillin (200 mg/kg) intragastrically once daily for 5 consecutive days to deplete the gut microbiota. Faecal samples of mice were freshly collected to detect bacterial DNA load and plated onto blood agar plates and cultured aerobically and anaerobically to confirm whether the gut microbiota was successfully depleted following ABX treatment. In the evening of day 5, food was removed, but mice had free access to water. Fresh faeces originating from multiple UOX^−/−^ littermate rats or WT littermate rats were collected under sterile conditions, combined and resuspended in pre-cooled sterile PBS (200 mg faeces/ml), and centrifuged for supernatant with 1000 rpm for 5 min at 4 °C. This step was repeated twice. The final bacterial suspension was mixed with an equal volume of 25% sterile glycerol to a final concentration of 12.5% and then stored at −80 °C until transplantation. An amount of 200 μl of bacterial suspension was transplanted into each recipient mice by gavage for three consecutive days. After the last gavage, mice were returned to clean cages and had free access to food and water. Inoculation was repeated twice a week to reinforce microbiota transplantation. Two weeks post-FMT, mice were subjected to renal ischemia/reperfusion surgery.

### Mouse models of I/R injury

Renal I/R surgery was performed by transiently clamping the unilateral renal artery. Mice were anaesthetized with a mixture of ketamine (50 mg/kg) and xylazine (7.5 mg/kg). After a laparotomy was performed, both kidneys were exposed. Subsequently, the renal pedicles were clamped using atraumatic vascular clamps for 30 min, followed by reperfusion for 24 h on a heated blanket. Twenty-four hours after reperfusion, the mice were sacrificed under anaesthesia to collect kidney samples for the next assessment.

### Statistical analyses

The data were analysed using the SPSS (Version 20.0, Chicago, USA) and R software and presented as mean ± SEM. After the data were tested for normality, two-tailed Student’s *t*-tests were used to compare two groups. For the comparisons of more than two groups, a one-way analysis of variance (ANOVA) was used, followed by the post hoc LSD test for multiple comparisons. The relationship between different variables was analysed by Pearson’s correlation, and its *p*-value was also calculated using the R software. A *p*-value of < 0.05 was considered to be statistically significant.

### Supplementary Information

Additional file 1: Figure S1. The effects of HUA-induced by UOX knockout on the food intake, water intake, and survival of rats. (A) Food intake of 8 week old rats (*n*=5). (B) Water intake of 8 week old rats (*n*=5). (C) Survival of rats in a 24-week observation period (*n*=30). Data are represented as mean±SEM. Statistical comparison was performed using two-tailed unpaired Student’s t tests or Kaplan-Meier analysis. **p<0.01. Figure S2. The effects of HUA on the microbial diversity and composition of rats. (A) ACE index (*n*=6). (B) Shannon index (*n*=6). (C) Chao1 index (*n*=6). (D) Relative abundance profile at a phylum level (*n*=6). (E) Relative abundance profile at a genus level (*n*=6). Data are represented as mean±SEM. Statistical comparison was performed using Wilcox rank-sum tests. Figure S3. Random permutation test with 200 permutations to evaluate the robustness of the OPLS-DA model. Figure S4. Heatmap of different metabolites in the kidneys of WT and UOX^-/-^rats. Figure S5. Pearson correlation between metabolites and renal function parameters. Red indicates a positive correlation while blue indicates a negative correlation. *p<0.05; **p<0.01. Figure S6. Correlation network among microbes, metabolites and renal injury parameters (Pearson coefficient>0.5 and *p*<0.05). Red indicates a positive correlation while blue indicates a negative correlation. Figure S7. ABX treatment successfully depleted the intestinal microbiota of mice. (A) Bacterial load in the faeces of control or ABX-treated mice (*n*=5). (B) Representative images of blood agar plating of faeces from control and ABX-treated mice. Data are represented as mean±SEM. Statistical comparison was performed using two-tailed unpaired Student’s t tests. **p<0.01. Figure S8. Function associated with the tryptophan metabolism were increased in HUA microbiota recipient mice after FMT. (A) Shannon index (*n*=4). (B) PCoA analysis using Bray-Curtis distances between FMTwt_sham and FMTuox_sham groups (*n*=4). (C) PCoA analysis using Bray-Curtis distances between FMTwt_IR and FMTuox_IR groups (*n*=4). (D) The relative abundance profile at a phylum level (*n*=4). (E) The relative abundance profile at a genus level (*n*=4). (F) Predicted KEGG functional pathway differences between FMTwt_sham and FMTuox_sham groups. (G) Predicted KEGG functional pathway differences between FMTwt_IR and FMTuox_IR groups. (H) Levels of serum IS in recipient mice after I/R (*n*=5). Data are represented as mean±SEM. Statistical comparison was performed using Wilcox rank-sum tests or two-tailed unpaired Student’s t tests. *p<0.05.

Additional file 2. The original data of targeted metabolomics to investigate the kidney metabolomic profiling between the UOX^-/-^and WT rats.

## Data Availability

No datasets were generated or analysed during the current study.

## Abbreviations

HUA: Hyperuricaemia
UOX: Uricase
WT: Wild type
CKD: Chronic kidney disease
AKI: Acute kidney injury
NLRP3: Nod-like receptor protein 3
UA: Uric acid
SCR: Serum creatinine
BUN: Blood urea nitrogen
KIM-1: Kidney injury molecule 1
NGAL: N-acetyl-b-D-glucosaminidase
RBP: Retinol-binding protein
β2-MG: β2-microglobulin
CysC: Cystatin C
LDA: Linear discriminant analysis
PCoA: Principal co-ordinates analysis
IS: Indoxyl sulphate
PC: p-Cresol
PCS: p-Cresyl sulphate
HA: Hippuric acid
PAGln: Phenylacetylglutamine
PAGly: Phenylacetylglycine
KA: Kynurenic acid
KYN: Kynurenine
QA: Quinolinic acid
MG: Methylguanidine
TMAO: Trimethylamine N-oxide
ABX: Antibiotics
FMT: Faecal microbiota transplantation
I/R: Ischemia/reperfusion
LPS: Lipopolysaccharide
H&E: Haematoxylin and eosin
PAS: Periodic acid-Schiff
AB: Alcian blue

## References

[1] Chen-Xu M, Yokose C, Rai SK, Pillinger MH, Choi HK (2019). Contemporary prevalence of gout and hyperuricemia in the United States and decadal trends: the National Health and Nutrition Examination Survey, 2007–2016. Arthritis Rheumatol.

[2] Zhang M, Zhu X, Wu J, Huang Z, Zhao Z, Zhang X (2021). Prevalence of hyperuricemia among chinese adults: findings from two nationally representative cross-sectional surveys in 2015–16 and 2018–19. Front Immunol.

[3] Dalbeth N, Gosling AL, Gaffo A, Abhishek A (2021). Gout. Lancet.

[4] Mortada I (2017). Hyperuricemia, type 2 diabetes mellitus, and hypertension: an emerging association. Curr Hypertens Rep.

[5] Shi Q, Wang R, Zhang H, Shan Y, Ye M, Jia B (2021). Association between serum uric acid and cardiovascular disease risk factors in adolescents in America: 2001–2018. PLoS One.

[6] Srivastava A, Kaze AD, Mcmullan CJ, Isakova T, Waikar SS (2018). Uric acid and the risks of kidney failure and death in individuals with CKD. Am J Kidney Dis.

[7] Chou YC, Kuan JC, Yang T, Chou WY, Hsieh PC, Bai CH (2015). Elevated uric acid level as a significant predictor of chronic kidney disease: a cohort study with repeated measurements. J Nephrol.

[8] El RR, Tallima H (2017). Physiological functions and pathogenic potential of uric acid: a review. J Adv Res.

[9] Jung SW, Kim SM, Kim YG, Lee SH, Moon JY (2020). Uric acid and inflammation in kidney disease. Am J Physiol Renal Physiol.

[10] Chen YY, Chen DQ, Chen L, Liu JR, Vaziri ND, Guo Y (2019). Microbiome-metabolome reveals the contribution of gut-kidney axis on kidney disease. J Transl Med.

[11] Wang H, Ainiwaer A, Song Y, Qin L, Peng A, Bao H (2023). Perturbed gut microbiome and fecal and serum metabolomes are associated with chronic kidney disease severity. Microbiome.

[12] Graboski AL, Redinbo MR. Gut-derived protein-bound uremic toxins. Toxins (Basel). 2020;12(9):590. 10.3390/toxins12090590 PMC7551879 32932981

[13] Lau WL, Savoj J, Nakata MB, Vaziri ND (2018). Altered microbiome in chronic kidney disease: systemic effects of gut-derived uremic toxins. Clin Sci (Lond).

[14] Xu D, Lv Q, Wang X, Cui X, Zhao P, Yang X (2019). Hyperuricemia is associated with impaired intestinal permeability in mice. Am J Physiol Gastrointest Liver Physiol.

[15] Guo Z, Zhang J, Wang Z, Ang KY, Huang S, Hou Q (2016). Intestinal microbiota distinguish gout patients from healthy humans. Sci Rep.

[16] Liu X, Lv Q, Ren H, Gao L, Zhao P, Yang X (2020). The altered gut microbiota of high-purine-induced hyperuricemia rats and its correlation with hyperuricemia. PeerJ.

[17] Wei J, Zhang Y, Dalbeth N, Terkeltaub R, Yang T, Wang Y (2022). Association between gut microbiota and elevated serum urate in two independent cohorts. Arthritis Rheumatol..

[18] Andrade-Oliveira V, Foresto-Neto O, Watanabe I, Zatz R, Camara N (2019). Inflammation in renal diseases: new and old players. Front Pharmacol.

[19] Rook GA (2013). Regulation of the immune system by biodiversity from the natural environment: an ecosystem service essential to health. Proc Natl Acad Sci U S A.

[20] Vilaysane A, Chun J, Seamone ME, Wang W, Chin R, Hirota S (2010). The NLRP3 inflammasome promotes renal inflammation and contributes to CKD. J Am Soc Nephrol.

[21] Zheng Z, Xu K, Li C, Qi C, Fang Y, Zhu N (2021). NLRP3 associated with chronic kidney disease progression after ischemia/reperfusion-induced acute kidney injury. Cell Death Discov.

[22] Mulay SR (2019). Multifactorial functions of the inflammasome component NLRP3 in pathogenesis of chronic kidney diseases. Kidney Int.

[23] He Y, Hara H, Nunez G (2016). Mechanism and regulation of NLRP3 inflammasome activation. Trends Biochem Sci.

[24] Zaki MH, Lamkanfi M, Kanneganti TD (2011). The Nlrp3 inflammasome: contributions to intestinal homeostasis. Trends Immunol.

[25] Mihajlovic M, Krebber MM, Yang Y, Ahmed S, Lozovanu V, Andreeva D, et al. Protein-bound uremic toxins induce reactive oxygen species-dependent and inflammasome-mediated IL-1beta production in kidney proximal tubule cells. Biomedicines. 2021;9(10):1326. 10.3390/biomedicines9101326 PMC8533138 34680443

[26] Zhao N, Chen QG, Chen X, Liu XT, Geng F, Zhu MM (2023). Intestinal dysbiosis mediates cognitive impairment via the intestine and brain NLRP3 inflammasome activation in chronic sleep deprivation. Brain Behav Immun.

[27] Lu J, Hou X, Yuan X, Cui L, Liu Z, Li X (2018). Knockout of the urate oxidase gene provides a stable mouse model of hyperuricemia associated with metabolic disorders. Kidney Int.

[28] Rukavina MN, Kouyoumdzian NM, Choi MR (2020). Gut microbiota and chronic kidney disease: evidences and mechanisms that mediate a new communication in the gastrointestinal-renal axis. Pflugers Arch.

[29] Lim YJ, Sidor NA, Tonial NC, Che A, Urquhart BL. Uremic toxins in the progression of chronic kidney disease and cardiovascular disease: mechanisms and therapeutic targets. Toxins (Basel). 2021;13(2):142. 10.3390/toxins13020142 PMC7917723 33668632

[30] Song S, Lou Y, Mao Y, Wen X, Fan M, He Z (2022). Alteration of gut microbiome and correlated amino acid metabolism contribute to hyperuricemia and Th17-driven inflammation in Uox-KO mice. Front Immunol.

[31] Wang X, Yang S, Li S, Zhao L, Hao Y, Qin J (2020). Aberrant gut microbiota alters host metabolome and impacts renal failure in humans and rodents. Gut.

[32] Pan L, Han P, Ma S, Peng R, Wang C, Kong W (2020). Abnormal metabolism of gut microbiota reveals the possible molecular mechanism of nephropathy induced by hyperuricemia. Acta Pharm Sin B.

[33] Feng YL, Cao G, Chen DQ, Vaziri ND, Chen L, Zhang J (2019). Microbiome-metabolomics reveals gut microbiota associated with glycine-conjugated metabolites and polyamine metabolism in chronic kidney disease. Cell Mol Life Sci.

[34] Wu IW, Lin CY, Chang LC, Lee CC, Chiu CY, Hsu HJ (2020). Gut microbiota as diagnostic tools for mirroring disease progression and circulating nephrotoxin levels in chronic kidney disease: discovery and validation study. Int J Biol Sci.

[35] Li G, Young KD (2013). Indole production by the tryptophanase TnaA in Escherichia coli is determined by the amount of exogenous tryptophan. Microbiology (Reading).

[36] Lobel L, Cao YG, Fenn K, Glickman JN, Garrett WS (2020). Diet posttranslationally modifies the mouse gut microbial proteome to modulate renal function. Science.

[37] Fu BC, Hullar M, Randolph TW, Franke AA, Monroe KR, Cheng I (2020). Associations of plasma trimethylamine N-oxide, choline, carnitine, and betaine with inflammatory and cardiometabolic risk biomarkers and the fecal microbiome in the Multiethnic Cohort Adiposity Phenotype Study. Am J Clin Nutr.

[38] Kim RB, Morse BL, Djurdjev O, Tang M, Muirhead N, Barrett B (2016). Advanced chronic kidney disease populations have elevated trimethylamine N-oxide levels associated with increased cardiovascular events. Kidney Int.

[39] Zeng Y, Guo M, Fang X, Teng F, Tan X, Li X (2021). Gut microbiota-derived trimethylamine N-oxide and kidney function: a systematic review and meta-analysis. Adv Nutr.

[40] Margiotta E, Miragoli F, Callegari ML, Vettoretti S, Caldiroli L, Meneghini M (2020). Gut microbiota composition and frailty in elderly patients with chronic kidney disease. PLoS One.

[41] Devlin AS, Marcobal A, Dodd D, Nayfach S, Plummer N, Meyer T (2016). Modulation of a circulating uremic solute via rational genetic manipulation of the gut microbiota. Cell Host Microbe.

[42] Mutsaers HA, Stribos EG, Glorieux G, Vanholder R, Olinga P (2015). Chronic kidney disease and fibrosis: the role of uremic retention solutes. Front Med (Lausanne).

[43] Niwa T, Shimizu H (2012). Indoxyl sulfate induces nephrovascular senescence. J Ren Nutr.

[44] Niwa T (2010). Indoxyl sulfate is a nephro-vascular toxin. J Ren Nutr.

[45] Huang Y, Zhou J, Wang S, Xiong J, Chen Y, Liu Y (2020). Indoxyl sulfate induces intestinal barrier injury through IRF1-DRP1 axis-mediated mitophagy impairment. Theranostics.

[46] Brito JS, Borges NA, Anjos J, Nakao LS, Stockler-Pinto MB, Paiva BR (2019). Aryl hydrocarbon receptor and uremic toxins from the gut microbiota in chronic kidney disease patients: is there a relationship between them?. Biochemistry.

[47] Meijers BK, Claes K, Bammens B, de Loor H, Viaene L, Verbeke K (2010). p-Cresol and cardiovascular risk in mild-to-moderate kidney disease. Clin J Am Soc Nephrol.

[48] Bammens B, Evenepoel P, Keuleers H, Verbeke K, Vanrenterghem Y (2006). Free serum concentrations of the protein-bound retention solute p-cresol predict mortality in hemodialysis patients. Kidney Int.

[49] Poveda J, Sanchez-Nino MD, Glorieux G, Sanz AB, Egido J, Vanholder R (2014). p-Cresyl sulphate has pro-inflammatory and cytotoxic actions on human proximal tubular epithelial cells. Nephrol Dial Transplant.

[50] Gryp T, Vanholder R, Vaneechoutte M, Glorieux G. p-Cresyl sulfate. Toxins (Basel). 2017;9(2):52. 10.3390/toxins9020052 PMC5331431 28146081

[51] Sun CY, Chang SC, Wu MS (2012). Suppression of Klotho expression by protein-bound uremic toxins is associated with increased DNA methyltransferase expression and DNA hypermethylation. Kidney Int.

[52] Zhu JZ, Zhang J, Yang K, Du R, Jing YJ, Lu L (2012). P-cresol, but not p-cresylsulphate, disrupts endothelial progenitor cell function in vitro. Nephrol Dial Transplant.

[53] Nemet I, Saha PP, Gupta N, Zhu W, Romano KA, Skye SM (2020). a cardiovascular disease-linked gut microbial metabolite acts via adrenergic receptors. Cell.

[54] Poesen R, Claes K, Evenepoel P, de Loor H, Augustijns P, Kuypers D (2016). Microbiota-derived phenylacetylglutamine associates with overall mortality and cardiovascular disease in patients with CKD. J Am Soc Nephrol.

[55] Sun B, Wang X, Liu X, Wang L, Ren F, Wang X, et al. Hippuric acid promotes renal fibrosis by disrupting redox homeostasis via facilitation of NRF2-KEAP1-CUL3 interactions in chronic kidney disease. Antioxidants (Basel). 2020;9(9):783. 10.3390/antiox9090783 PMC7555723 32854194

[56] Fujiyoshi N, Feketeova E, Lu Q, Xu DZ, Hasko G, Deitch EA (2006). Amiloride moderates increased gut permeability and diminishes mesenteric lymph-mediated priming of neutrophils in trauma/hemorrhagic shock. Surgery.

[57] Gong S, Lan T, Zeng L, Luo H, Yang X, Li N (2018). Gut microbiota mediates diurnal variation of acetaminophen induced acute liver injury in mice. J Hepatol.

