# Supplementary material for: Gut microbiota dysbiosis in hyperuricaemia promotes renal injury through the activation of NLRP3 inflammasome
Source: Microbiome. 2024 Jun 21;12:109. doi: 10.1186/s40168-024-01826-9 (PMC11191305; doi:10.1186/s40168-024-01826-9)
Supplement: Supplementary file 2 — Additional file 1: Figure S1. The effects of HUA-induced by UOX knockout on the food intake, water intake, and survival of rats. (A) Food intake of 8 week old rats (n=5). (B) Water intake of 8 week old rats (n=5). (C) Survival of rats in a 24-week observation period (n=30). Data are represented as mean±SEM. Statistical comparison was performed using two-tailed unpaired Student’s t tests or Kaplan-Meier analysis. **p<0.01. Figure S2. The effects of HUA on the microbial diversity and composition of rats. (A) ACE index (n=6). (B) Shannon index (n=6). (C) Chao1 index (n=6). (D) Relative abundance profile at a phylum level (n=6). (E) Relative abundance profile at a genus level (n=6). Data are represented as mean±SEM. Statistical comparison was performed using Wilcox rank-sum tests. Figure S3. Random permutation test with 200 permutations to evaluate the robustness of the OPLS-DA model. Figure S4. Heatmap of different metabolites in the kidneys of WT and UOX-/-rats. Figure S5. Pearson correlation between metabolites and renal function parameters. Red indicates a positive correlation while blue indicates a negative correlation. *p<0.05; **p<0.01. Figure S6. Correlation network among microbes, metabolites and renal injury parameters (Pearson coefficient>0.5 and p<0.05). Red indicates a positive correlation while blue indicates a negative correlation. Figure S7. ABX treatment successfully depleted the intestinal microbiota of mice. (A) Bacterial load in the faeces of control or ABX-treated mice (n=5). (B) Representative images of blood agar plating of faeces from control and ABX-treated mice. Data are represented as mean±SEM. Statistical comparison was performed using two-tailed unpaired Student’s t tests. **p<0.01. Figure S8. Function associated with the tryptophan metabolism were increased in HUA microbiota recipient mice after FMT. (A) Shannon index (n=4). (B) PCoA analysis using Bray-Curtis distances between FMTwt_sham and FMTuox_sham groups (n=4). (C) PCoA analysis usin [file 40168_2024_1826_MOESM1_ESM.docx]

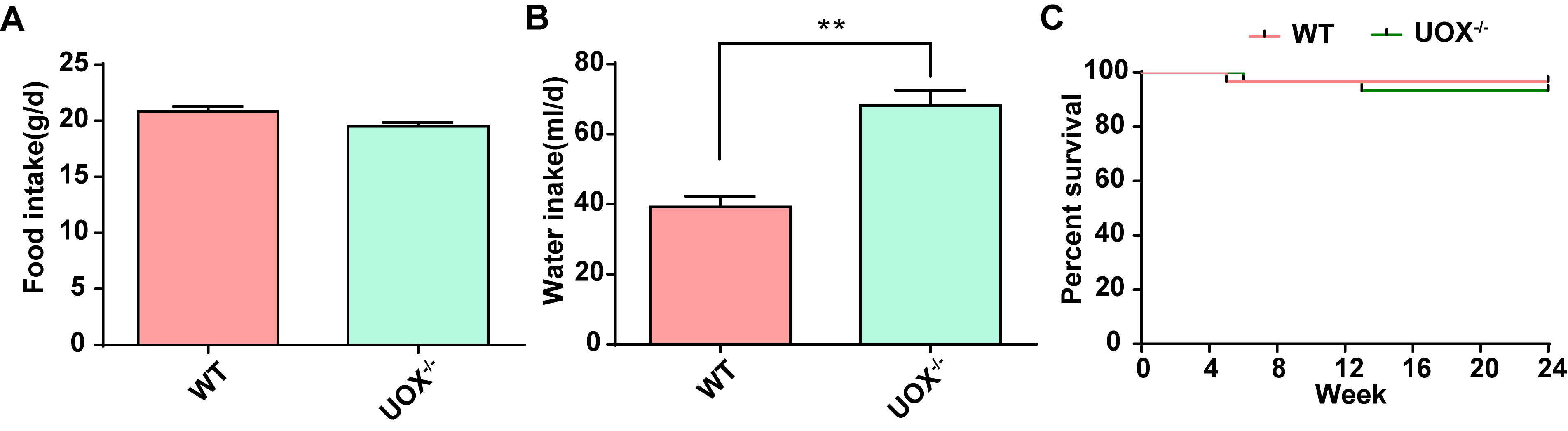


**Fig.S1 The effects of HUA-induced by UOX knockout on the food intake, water intake, and survival of rats.** **A** Food intake of 8 week old rats (n=5). **B** Water intake of 8 week old rats (n=5). **C** Survival of rats in a 24-week observation period (n=30). Data are represented as mean±SEM. Statistical comparison was performed using two-tailed unpaired Student’s t tests or Kaplan-Meier analysis. ***p*<0.01.


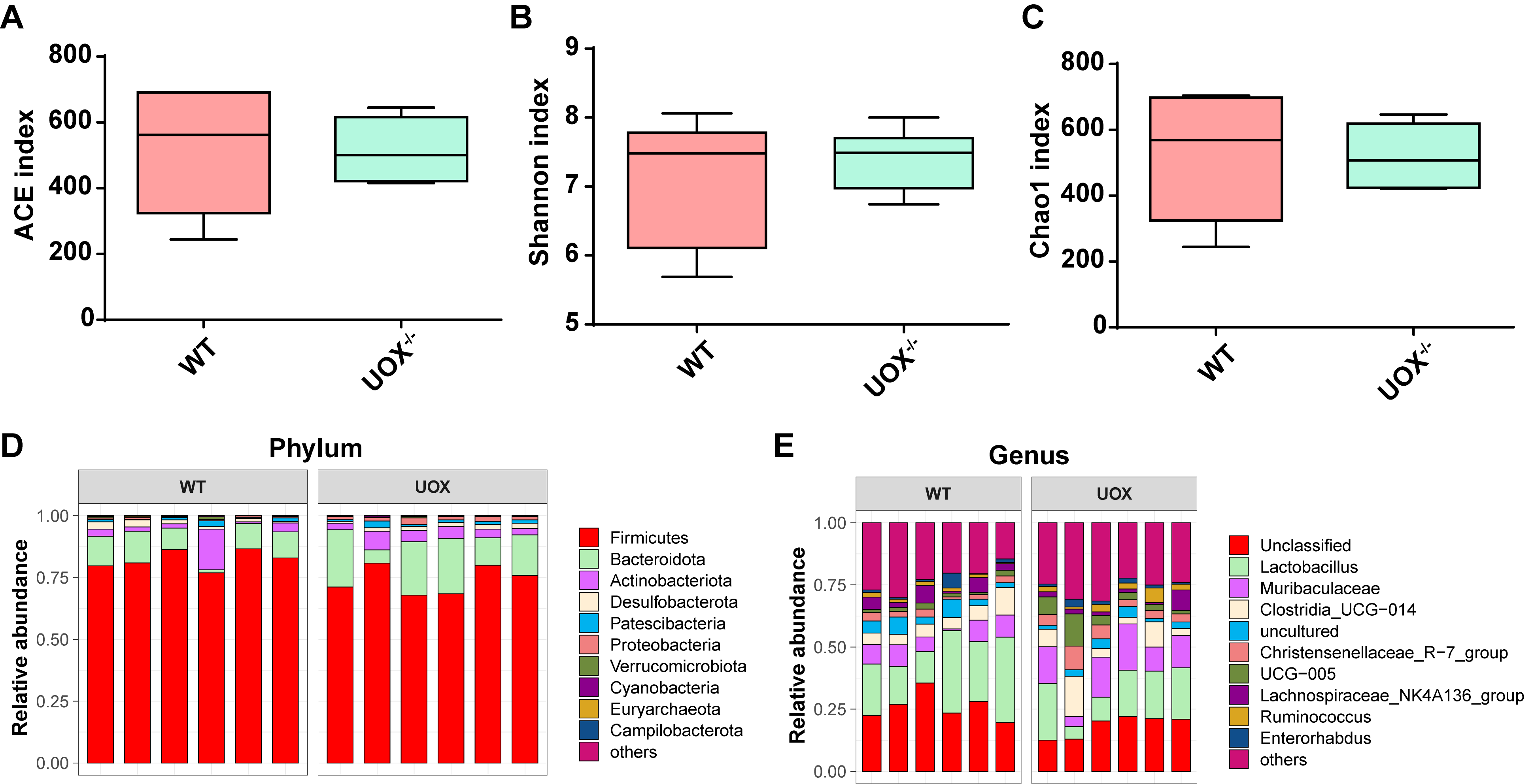


**Fig.S2 The effects of HUA on the microbial diversity and composition of rats. A** ACE index (n=6). **B** Shannon index (n=6). **C** Chao1 index (n=6). **D** The relative abundance profile at a phylum level (n=6). **E** The relative abundance profile at a genus level (n=6). Data are represented as mean±SEM. Statistical comparison was performed using Wilcox rank-sum tests.


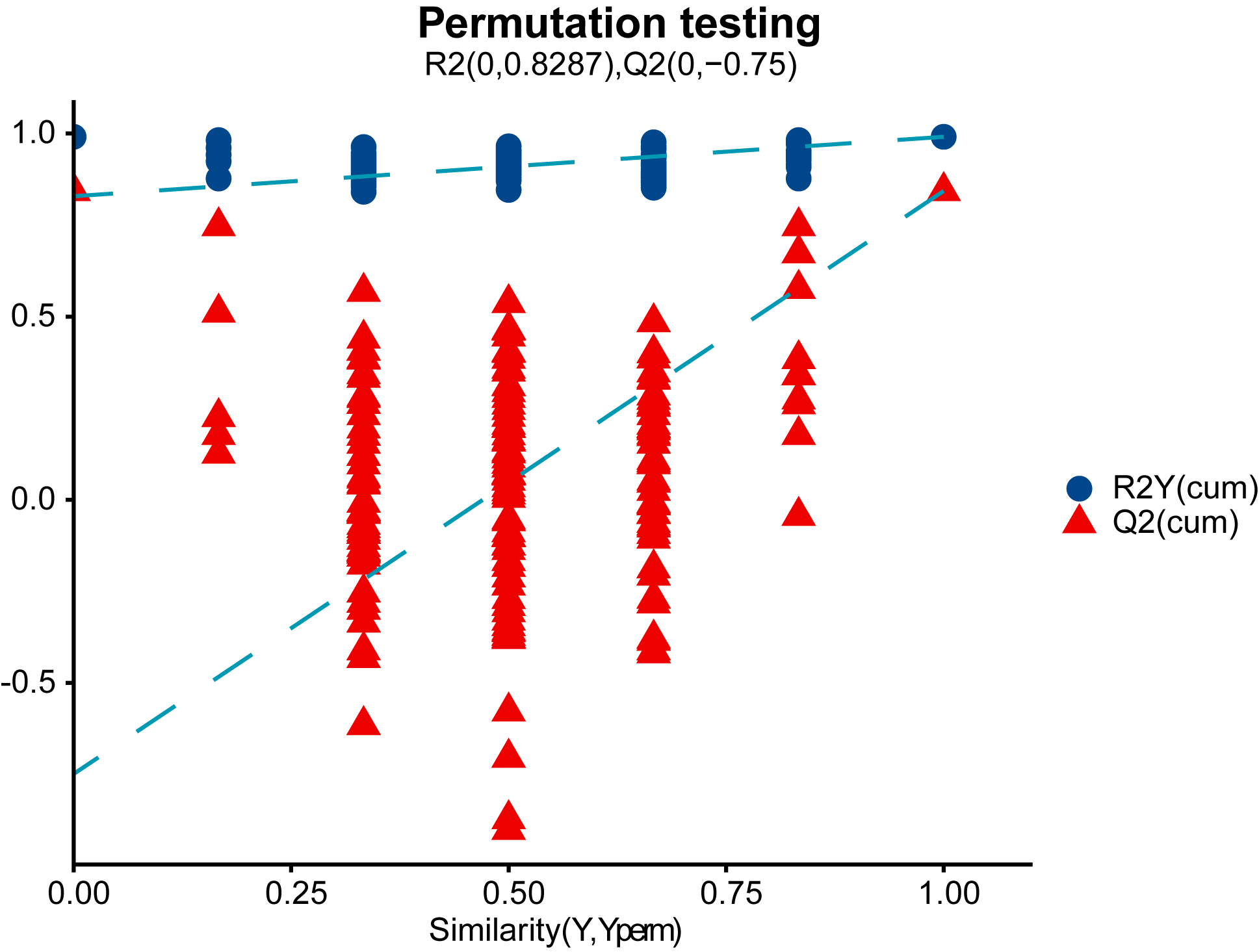


**Fig.S3 Random permutation test with 200 permutations to evaluate the robustness of the OPLS-DA model.**


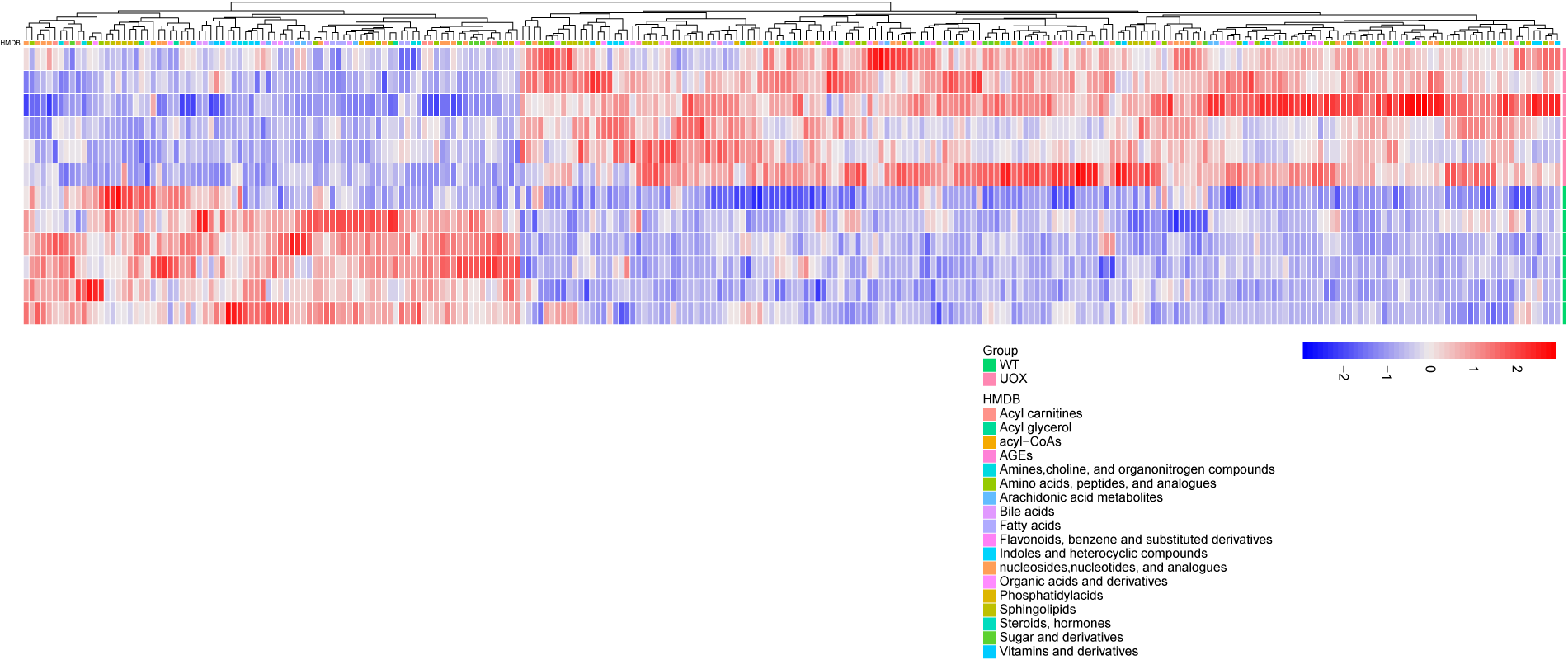


**Fig.S4 Heatmap of differentmetabolites in the kidneys of WT and UOX^-/-^ rats.**

**Fig.S5 Pearson correlation between metabolites and renal function parameters.** Red indicates a positive correlation while blue indicates a negative correlation. **p*<0.05; ***p*<0.01.

**Fig.S6 Correlation network among microbes, metabolites and renal injury parameters (Pearson coefficient >0.5 and *p*<0.05).** Red color indicates a positive correlation while tblue indicates a negative correlation.

**Fig.S7 ABX treatment successfully depleted the intestinal microbiota of mice.** **A** Bacterial load in the faeces of control or ABX-treated mice (n=5). **B** Representative images of blood agar plating of feces from control and ABX-treated mice. Data are represented as mean±SEM. Statistical comparison was performed using two-tailed unpaired Student’s t tests. ***p*<0.01.

**Fig.S8 Function associated with tryptophan metabolism were increased in HUA microbiota recipient mice after FMT.** **A** Shannon index (n=4). **B** PCoA analysis using Bray-Curtis distances between FMTwt_sham and FMTuox_sham groups (n=4). **C** PCoA analysis using Bray-Curtis distances between FMTwt_IR and FMTuox_IR groups (n=4). **D** The relative abundance profile at a phylum level (n=4). **E** The relative abundance profile at a genus level (n=4). **F** Predicted KEGG functional pathway differences between FMTwt_sham and FMTuox_sham groups. **G** Predicted KEGG functional pathway differences between FMTwt_IR and FMTuox_IR groups. **H** Levels of serum IS in recipient mice after I/R (n=5). Data are represented as mean±SEM. Statistical comparison was performed using Wilcox rank-sum test or two-tailed unpaired Student’s t test. **p*<0.05.
